# Increased expression of the PI3K catalytic subunit p110δ underlies elevated S6 phosphorylation and protein synthesis in an individual with autism from a multiplex family

**DOI:** 10.1186/s13229-015-0066-4

**Published:** 2016-01-14

**Authors:** Ashwini C. Poopal, Lindsay M. Schroeder, Paul S. Horn, Gary J. Bassell, Christina Gross

**Affiliations:** Department of Cell Biology, Emory University Medical School, 615 Michael Street, Atlanta, GA 30322 USA; Division of Neurology, Cincinnati Children’s Hospital Medical Center, 3333 Burnet Avenue, Cincinnati, OH 45229 USA; Division of Biostatistics and Epidemiology, Cincinnati Children’s Hospital Medical Center, 3333 Burnet Avenue, Cincinnati, OH 45229 USA

**Keywords:** PI3K/mTOR signaling, p110δ, Autism, Biomarker, S6 phosphorylation, IC87114

## Abstract

**Background:**

Dysfunctions in the PI3K/mTOR pathway have gained a lot of attention in autism research. This was initially based on the discovery of several monogenic autism spectrum disorders with mutations or defects in PI3K/mTOR signaling components. Recent genetic studies corroborate that defective PI3K/mTOR signaling might be a shared pathomechanism in autism disorders of so far unknown etiology, but functional molecular analyses in human cells are rare. The goals of this study were to perform a functional screen of cell lines from patients with idiopathic autism for defects in PI3K/mTOR signaling, to test if further functional analyses are suitable to detect underlying molecular mechanisms, and to evaluate this approach as a biomarker tool to identify therapeutic targets.

**Methods:**

We performed phospho-S6- and S6-specific ELISA experiments on 21 lymphoblastoid cell lines from the AGRE collection and on 37 lymphoblastoid cell lines from the Simons Simplex Collection and their healthy siblings. Cell lines from one individual with increased S6 phosphorylation and his multiplex family were analyzed in further detail to identify upstream defects in PI3K signaling associated with autism diagnosis.

**Results:**

We detected significantly increased S6 phosphorylation in 3 of the 21 lymphoblastoid cell lines from AGRE compared to a healthy control and in 1 of the 37 lymphoblastoid cell lines from the Simons Simplex Collection compared to the healthy sibling. Further analysis of cells from one individual with elevated S6 phosphorylation showed increased expression of the PI3K catalytic subunit p110δ, which was also observed in lymphoblastoid cells from other autistic siblings but not unaffected members in his multiplex family. The p110δ-selective inhibitor IC87114 reduced elevated S6 phosphorylation and protein synthesis in this cell line.

**Conclusions:**

Our results suggest that functional analysis of PI3K/mTOR signaling is a biomarker tool to identify disease-associated molecular defects that could serve as therapeutic targets in autism. Using this approach, we discovered impaired signaling and protein synthesis through the PI3K catalytic subunit p110δ as an underlying molecular defect and potential treatment target in select autism spectrum disorders. Increased p110δ activity was recently associated with schizophrenia, and our results suggest that p110δ may also be implicated in autism.

**Electronic supplementary material:**

The online version of this article (doi:10.1186/s13229-015-0066-4) contains supplementary material, which is available to authorized users.

## Background

The PI3K/mTOR pathway regulates a variety of functions throughout the body, including fundamental processes such as cell growth, differentiation and survival. In neurons, the PI3K/mTOR pathway was shown to regulate protein synthesis [1], to play a role in dendritic morphology and axon outgrowth and regeneration [2–4], and to be important for synaptic plasticity [5–7]. Mutations in this pathway are among the most abundant in cancer affecting virtually all organs of the body including the nervous system and particularly the brain. Several clinical trials have been initiated to test PI3K/mTOR antagonists for their efficacy to halt or reduce tumor growth [8], but more recently this pathway has also gained considerable attention for its dysregulation and potential applicability as a therapeutic target in other brain disorders, including epilepsy, schizophrenia, and autism spectrum disorders [9–13].

Several monogenic autism spectrum disorders have mutations leading to defects in the PI3K pathway, such as tuberous sclerosis (TS) [14], fragile X syndrome (FXS) [1, 15], neurofibromatosis 1 (NF1) [16], and brain disorders associated with phosphatase and tensin homolog (PTEN) mutations [17]. More recently, genome-wide association studies and analyses of chromosome copy number variations (CNVs) have revealed even more evidence for dysregulation of the PI3K/mTOR signaling complex in autism spectrum disorders [18–21]. Lastly, autism has been associated with brain overgrowth, cortical malformations, and aberrant growth trajectories [22], further suggesting that dysfunctions in the PI3K/mTOR pathway, an important regulator of cell growth, might contribute to the autism etiology. However, to what extent alterations in brain size and connectivity are a shared characteristic of autism remains controversial [23].

Together, these findings suggest that there is a considerable subgroup of autism spectrum disorders of unknown etiology with defects in PI3K/mTOR signaling, which could qualify for a therapeutic strategy targeted at this pathway. While genetic screens are important to identify potentially pathological mutations in the PI3K/mTOR pathway, they do not directly reveal the functional consequences and how signal transduction is affected by a certain mutation in human patient cells. Understanding the exact pathological mechanisms in PI3K/mTOR signaling, which may be the sum of several mutations as well as epigenetic factors, will be essential to identify efficient, disease-targeting therapies.

Here, we tested the hypothesis that functional defects in PI3K/mTOR signaling can be detected and utilized to identify potential therapeutic targets in lymphoblastoid cell lines from patients with idiopathic autism. In particular, we performed quantitative analyses of phosphorylation of the PI3K/mTOR downstream target S6 in patient cell lines from the Autism Genetic Research Exchange (AGRE) collection and in proband-sibling pairs from the Simons Simplex Collection (SSC). We identified several cell lines with increased S6 phosphorylation suggesting elevated upstream signaling. We confirmed the pathological relevance of increased S6 phosphorylation for one patient from the AGRE collection by showing that his unaffected sister did not have increased S6 phosphorylation. Analyses of upstream signaling defects and pharmacological rescue experiments using PI3K catalytic subunit-selective inhibitors suggested that increased expression and activity of the PI3K catalytic subunit p110δ, but not p110β, underlie increased S6 phosphorylation and protein synthesis in this autism patient cell line. This work supports the applicability of functional molecular screens to detect signal transduction defects in lymphoblastoid cell lines from patients with autism that could serve as therapeutic targets.

## Methods

### Lymphoblastoid cell lines

Lymphoblastoid cell lines were obtained from the Autism Genetic Research Exchange (AGRE; cell lines marked as A1-A21, all male and diagnosed with autism, and A4-F, -M, -S, -B1, -B2, -B3: multiplex family of A4, pedigree shown in Fig. 5a), the Simons Simplex Collection (SSC, cell lines marked as S1-37, including the individual with autism (*proband*) and the unaffected sibling, all male), and the Coriell Institute (Camden, NJ, USA; *CTR*, *CTR-2*). The institutional review boards of Emory University and Cincinnati Children’s Hospital Medical Center determined that this research was “exempt” and no IRB approval was required. Cell lines were grown in RPMI supplemented with 10 % fetal bovine serum and penicillin/streptavidin (100 U/ml) and kept at a cell density between 500,000 and 800,000 cells/ml. If cell lines differed in growth rates, as monitored by change of color of the media and cell density, they were discarded, and new batches were thawed. Cell lines were only kept in culture and used for experiments for up to 5 weeks. Experimental data were obtained from at least two separate frozen vials of cell lines.

### Antibodies and drugs

The phospho-S6 antibody (Ser235/236, #2211), S6 antibody (#2317), and p110α antibody (#4255) were obtained from Cell Signaling Technology. p110δ antibodies (#sc-7176, mab2687) were obtained from Santa Cruz Biotechnology and R&D Biosciences, respectively. The p110β antibody (#09-482) was obtained from EMD Millipore, the α-tubulin antibody (T6074) was obtained from Sigma-Aldrich, and the anti-puromycin antibody was purchased from the Developmental Studies Hybridoma Bank (University of Iowa). Secondary antibodies for Western blotting were obtained from VWR (horseradish peroxidase-coupled anti-mouse and anti-rabbit antibodies for ECL) or from Li-COR Biosciences (IRDye 680 RD donkey anti-mouse IgG and IRDye 800CW goat anti-rabbit IgG for infrared fluorescent Western blotting). Puromycin was purchased from Life Technologies, the p110δ inhibitor IC87114 was purchased from EMD Millipore, and the p110β inhibitor TGX-221 was purchased from Selleck Chemicals.

### S6- and phospho-S6 ELISAs

S6- and phospho-S6 ELISA kits were obtained from Cell Signaling Technology and run in triplicates and in parallel using 150 μg of protein per sample following the manufacturer’s protocol. Lymphoblastoid cell lines were lysed by sonication using the provided lysis buffer.

### PI3K activity assay

PI3K activity assays were performed using the PI3-kinase activity ELISA: Pico (Echelon Biosciences Incorporated) as described previously [24]. Briefly, lymphoblastoid cell lines were washed once with ice-cold PBS then lysed using sonication in ice-cold PI3K assay lysis buffer (50 mM Tris–HCl, pH 7.4, 40 mM NaCl, 1 mM EDTA, 0.5 % Triton X-100, 1.5 mM Na_3_VO_4_, 50 mM NaF, 10 mM sodium pyrophosphate, and 10 mM sodium glycerol phosphate, supplemented with proteinase inhibitors). One hundred micrograms of protein was used for subsequent immunoprecipitation with a p110δ-specific antibody (mab2687, R&D Biosciences). PI3K assays were performed as described by the manufacturer with the following modifications: kinase reactions were conducted for 3 h in 60 μl volume; the reaction contained 75 μM ATP, 1 mM DTT, and 15 μM phosphatidylinositol (3,4) biphosphate diC8 and was terminated with 2.4 mM EDTA. The amount of phosphatidylinositol-(3,4,5)-triphosphate generated during the reaction was quantified using defined standards.

### Western blotting

Equal amounts of protein lysates (prepared as described above) were resolved on SDS protein acrylamide gels, transferred to PVDF membranes, and detected with specific antibodies by enhanced chemiluminescence using standard methods and autoradiography (phospho-S6, S6, p110α, p110β, p110δ, Akt, tubulin) or by Odyssey infrared fluorescence Western blotting (Li-COR Biosciences) using the manufacturer’s blocking buffer (PBS) for blocking and antibody incubation (puromycin, Akt).

### Protein synthesis assay

Protein synthesis assays were performed using puromycinylation of nascent peptide chains as described previously [25, 26]. Briefly, equal amounts of cells (ca. 4–7 million) were resuspended in 7–8 ml of culturing media. Cells were treated with 1 nM IC87114, 1 μM TGX-221, or an equal amount of vehicle for 15 min, followed by incubation with 0.5 μg/ml puromycin for another 15 min. Cells were spun down and washed three times with ice-cold PBS and then lysed and analyzed by puromycin-specific Western blotting as described above. For quantification, signal intensities of the whole lanes spanning the entire molecular weight range of proteins were analyzed using ImageJ (*NIH*).

### Statistics

Statistical analyses were performed using SAS® version 9.3 (SAS Institute Inc., Cary, NC; Fig. 2 and Additional file 1: Figure S1d) and GraphPad Prism 6 (GraphPad Software Incorporated; all other figures). Phospho-S6 and S6 levels in AGRE cells were analyzed by one-way ANOVA followed by Dunnett’s post hoc analyses to compare each autistic individual to the healthy control. SSC cells were analyzed using a two-way ANOVA with family and disease status as fixed factors. Pairwise comparisons (proband to his healthy sibling) were controlled for type I errors by false discovery rate correction using an overall error rate equal to 0.05. All other data were analyzed by paired *t* tests, one-way ANOVA, or two-way ANOVA as appropriate followed by post hoc analyses (indicated in the text and figure legends). Bar diagrams and error bars illustrate means and standard error of the mean; *n* is indicated in each figure and/or figure legend.

## Results

### Identification of increased S6 phosphorylation in lymphoblastoid cell lines from individuals with autism

**Fig. 1:**
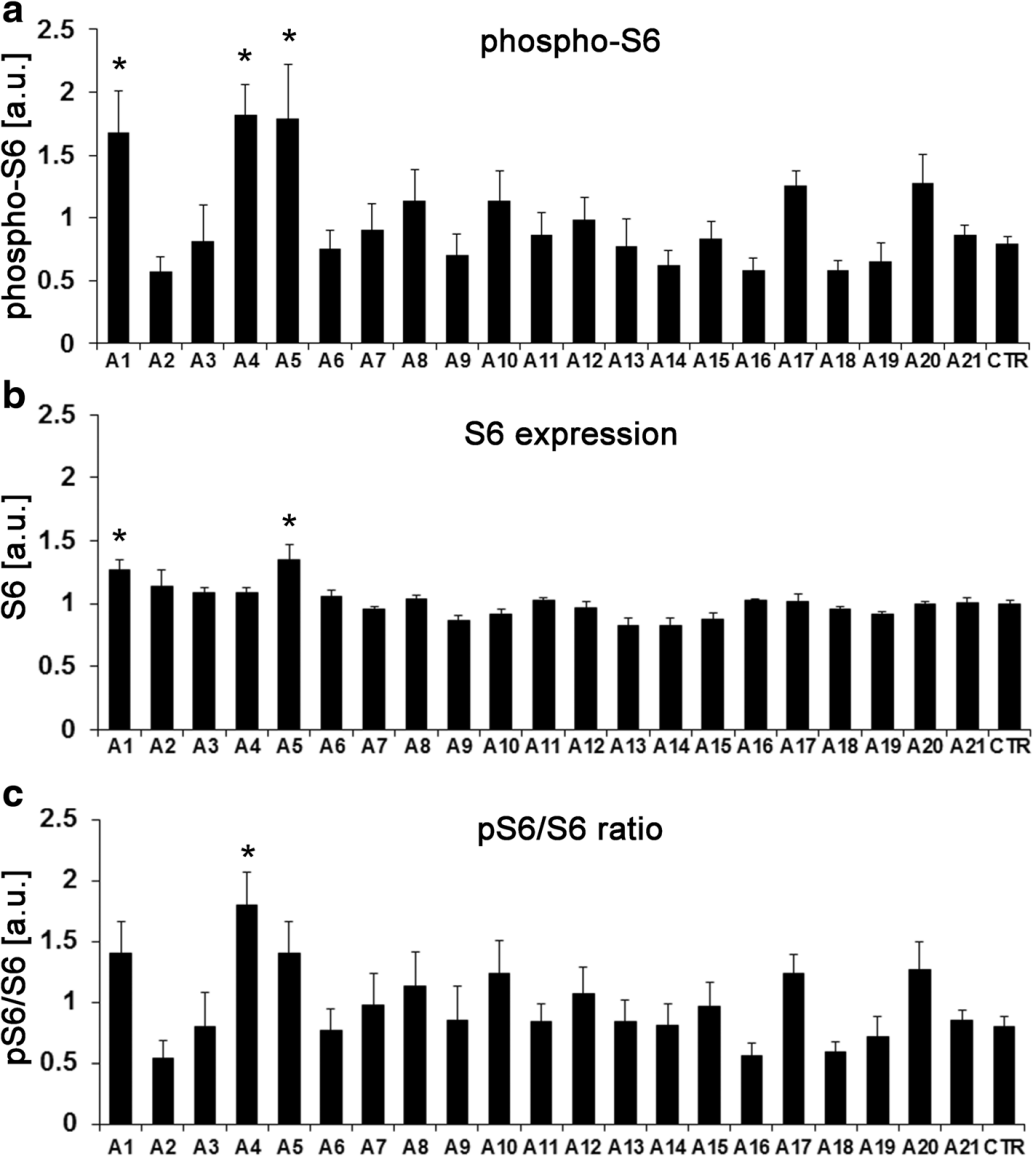
Phospho-S6- and S6-specific ELISAs on lymphoblastoid cell lines from patients with idiopathic autism from AGRE. **a** Phosphorylation of S6 is significantly increased in three cell lines (A1, A4, and A5) (one-way ANOVA *F*(21,85) = 3.5; *p* < 0.0001, Dunnett’s post hoc comparisons of autism cell lines to the unaffected control (*CTR*) *p(A1) = 0.041, *p(A4) = 0.011, *p(A5) = 0.015). **b** Expression of total S6 (phosphorylated and unphosphorylated) shows much less variability than phospho-S6. Two of the three cell lines with increased S6 phosphorylation also have increased S6 levels (A1 and A5) (one-way ANOVA *F*(21,85) = 5.468; *p* < 0.0001, Dunnett’s post hoc comparisons of autism cell lines to the unaffected control *p(A1) = 0.013, *p(A5) = 0.0003). **c** Phospho-S6/S6 ratios were significantly increased in cell line A4, suggesting an upstream signal transduction defect (one-way ANOVA *F*(21,85) = 2.34; *p* = 0.003, Dunnett’s post hoc comparisons of autism cell lines to the unaffected control *p(A4) = 0.014). *N* = 4, shown are means + SEM. List of *p* values for all pairwise comparisons in Additional file 2: Table S1a. Further control experiments are shown in Additional file 1: Figure S1a–c

**Fig. 2:**
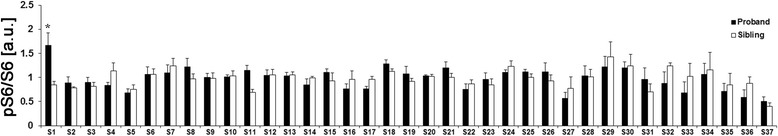
Phospho-S6/S6 ratios of lymphoblastoid cells from autistic individuals and their unaffected sibling from the SSC. ELISA analyses showed that one autistic individual had a significantly increased phospho-S6/S6 ratio compared to his healthy sibling (two-way ANOVA, family and disease status as fixed factors, disease status *F*(1,218) = 0.07, *p* = 0.79; family *F*(36,218) = 3.02, *p* < 0.0001; interaction *F*(36,218) = 1.24, *p* = 0.17; false discovery rate-corrected *p = 0.003). *N* = 3–4, shown are means + SEM. List of *p* values for all pairwise comparisons in Additional file 2: Table S1b. Further control experiments are described in Additional file 1: Figure S1d.

Dysregulated PI3K/mTOR signaling in the brain has been detected and successfully targeted to correct phenotypes in several mouse models of autism, including FXS [1, 27–31]. Defects in this signaling pathway might thus be a shared, targetable pathological mechanism in autism disorders of diverse etiologies. We and others have previously shown that altered PI3K/mTOR signaling, which contributes to neuronal dysfunction and autistic-like phenotypes, can be detected in peripheral cells from individuals with FXS, such as lymphoblastoid cell lines and fibroblasts [24, 32, 33]. To assess if abnormal PI3K/mTOR-mediated signaling as a shared molecular defect in autism is detectable in peripheral cell lines from humans with idiopathic autism, we analyzed lymphoblastoid cell lines from the Autism Genetic Research Exchange (AGRE) collection and the Simons Simplex Collection (SSCs). AGRE collects lymphocytes from autistic individuals from simplex and multiplex families with none, one, or several additional siblings diagnosed with autism, whereas the SSC contains lymphoblastoid cell lines from families with one autistic child that has at least one healthy sibling. S6 phosphorylation and S6 expression from 21 individuals with autism from AGRE were compared to an unaffected control using ELISA analyses (Fig. 1). Three out of 21 individuals showed significantly increased S6 phosphorylation compared to an unaffected control (Fig. 1a, one-way ANOVA *F*(21,85) = 3.5; *p* < 0.0001, Dunnett’s post hoc comparisons of autism cell lines to the unaffected control *p < 0.05). Two of these three also had significantly increased S6 expression, suggesting that increased phosphorylation was due to an overall increase in S6 (Fig. 1b, one-way ANOVA *F*(21,85) = 5.47; *p* < 0.0001, Dunnett’s post hoc comparisons of all autism cell lines to the unaffected control *p < 0.05). No differences in total S6 levels between any other cell line and the control cell line were detected. Consequently, one cell line (A4) had significantly increased pS6/S6 ratios suggesting a defect in the upstream signaling pathway (Fig. 1c, one-way ANOVA *F*(21,85) = 2.34; *p* = 0.003, Dunnett’s post hoc comparisons of all autism cell lines to the unaffected control *p = 0.013). A list of *p* values for all pairwise comparisons can be found in Additional file 2: Table S1a. Independent repeats of assays in the unaffected control used in Fig. 1 as well as experiments using an additional unaffected control line (*CTR-2*) supported the reproducibility of the assay results and showed that there was no significant difference in S6 phosphorylation in the two control lines (Additional file 1: Figure S1a–c). Nonetheless, differences in S6 phosphorylation could be due to autism-unrelated inherited genetic predispositions, and a lymphoblastoid cell line generated from a random, unrelated healthy individual might thus not be the ideal control. To address this issue, we followed a second approach, in which we took advantage of the SSC, a collection of lymphoblastoid cell lines from autism simplex families, in which for every individual with autism, at least one healthy sibling is available. Here, we directly compared every cell line from affected individuals with the cell line from the respective unaffected sibling. In 2 out of 37 proband-sibling pairs (S1 and S11), there was a consistent increase in the ratio of phospho-S6 to S6 in the proband compared to his healthy sibling in four repeats, with *p* < 0.05 for one pair (S1) after false discovery rate correction (Fig. 2, two-way-ANOVA, family and disease status as fixed factors; list of *p* values for all pairwise comparisons in Additional file 2: Table S1b). To further investigate whether differences in S6 phosphorylation could be due to autism-independent genetic predispositions in unrelated individuals, we compared phospho-S6/S6 ratios in the unaffected siblings. A one-way ANOVA showed a significant difference (*F*(36,109) = 1.7, *p* = 0.018), which was mainly driven by sibling S37 (see Additional file 1: Figure S1d, and associated figure legend). Of note, this was an effect of the family S37, as the phospho-S6/S6 ratio of the proband S37 was very low, too, and there was no difference between sibling S37 and the proband S37 (FDR-adjusted *p* = 0.919; Fig. 2 and Additional file 2: Table S1b). These results show that most unaffected controls have similar phospho-S6/S6 ratios (*CTR* and *CTR-2*, healthy siblings S1-S36). This data further supports the validity of our approach to directly compare autistic individuals to their healthy family members to account for effects of autism-unrelated genetic predispositions or environmental influences.

### Elevated expression and activity of the PI3K catalytic subunit p110δ in a cell line with increased S6 phosphorylation

**Fig. 3:**
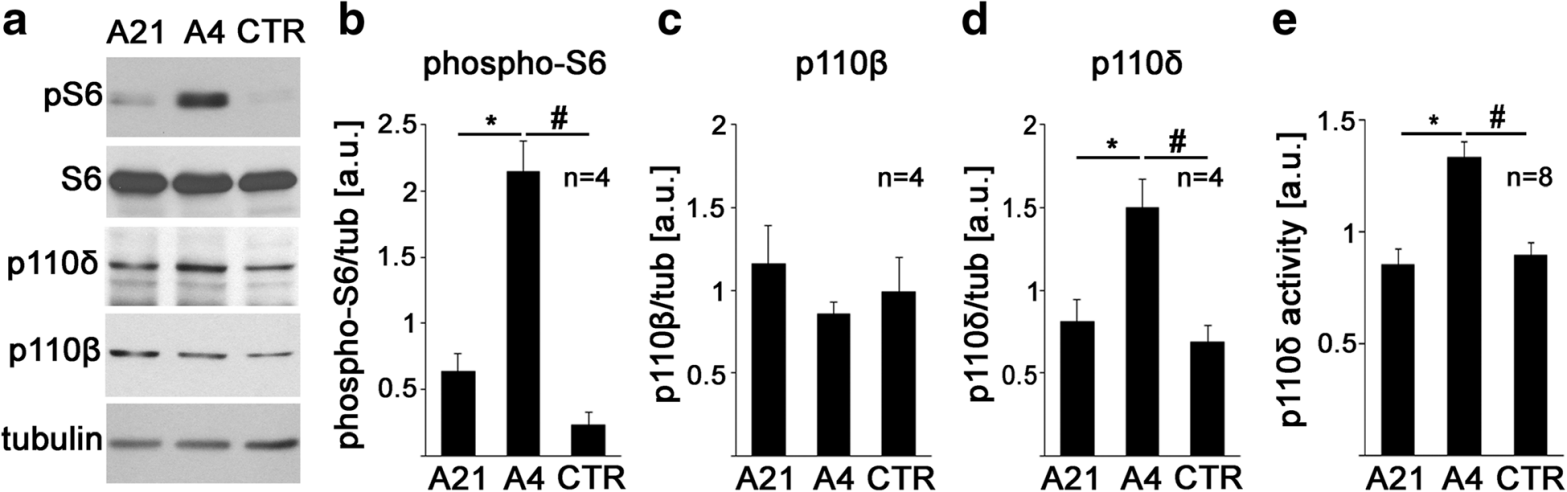
Elevated expression and activity of the PI3K catalytic subunit p110δ in an autism cell line with increased pS6/S6 ratio. **a** Example Western blots showing increased phosphorylation of S6, increased p110δ, but no changes in S6, p110β, or tubulin in cell line A4 compared to another autism cell line (A21) and an unaffected control (*CTR*). **b**–**d** Densitometric quantification of pS6- (**b**), p110β- (**c**), and p110δ- (**d**) specific Western blots shows significantly increased S6 phosphorylation and p110δ levels but no changes in p110β (*n* = 4, one-way ANOVAs with Tukey’s post hoc analyses; **b** *F*(2,9) = 35.36; *p* < 0.0001; *p = 0.0004, ^#^ *p* < 0.0001; **c** *F*(2,9) = 0.66; *p* = 0.54; **d** *F*(2,9) = 10.42; *p* = 0.0045; *p = 0.014, ^#^ *p* = 0.0056). All values were normalized to tubulin. **e** p110δ-associated PI3K activity is significantly increased in cell line A4 compared to cell line A21 and the unaffected control (*n* = 8, one-way ANOVA with Tukey’s post hoc analyses, *F*(2,21) = 14.73; *p* = 0.0001; *p = 0.0002, ^#^ *p* = 0.0006). Bar diagrams are means + SEM

**Fig. 4:**
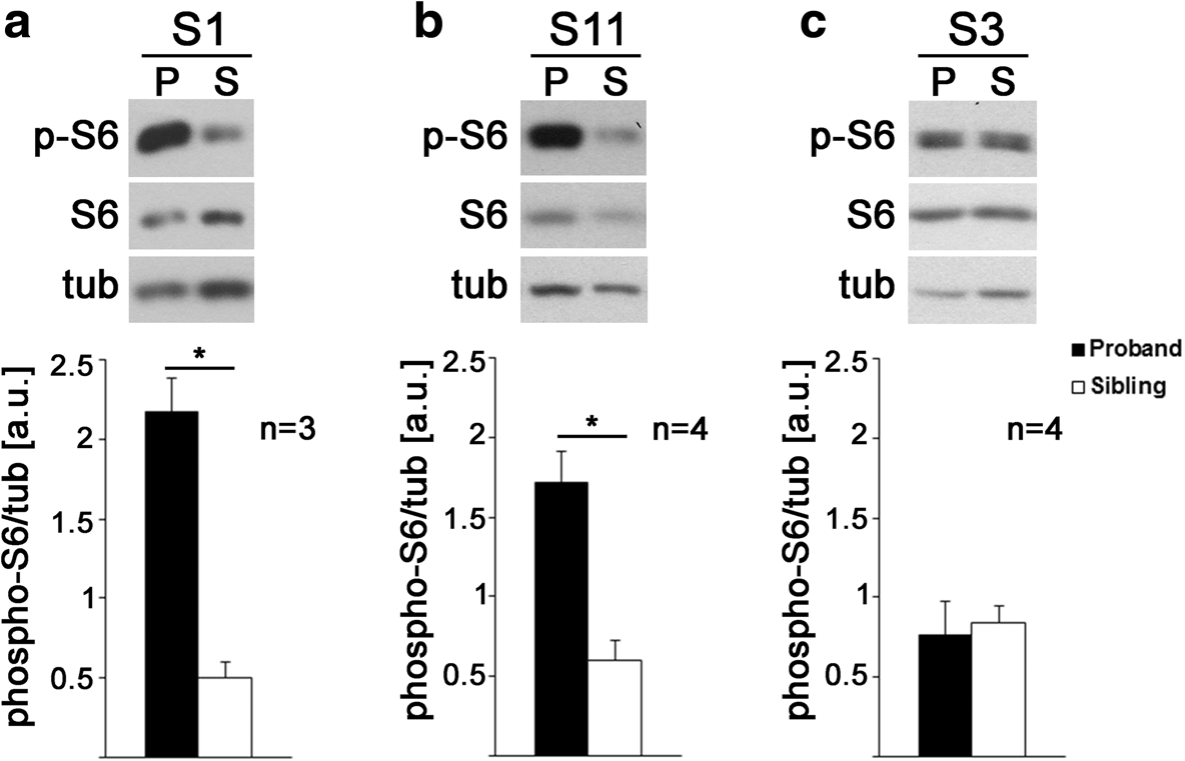
Western blot analyses confirm increased S6 phosphorylation in select SSC cell lines compared to their unaffected siblings. S6 phosphorylation was significantly increased in cell line S1 (**a**, *n* = 3, paired *t* test, *t*(2) = 10.7, *p* = 0.009) and S11 (**b**, *n* = 4, paired *t* test, *t*(3) = 10.39, *p* = 0.002) but not in S3 (**c**, *n* = 4, paired *t* test, *t*(3) = 0.72, *p* = 0.525), confirming ELISA results shown in Fig. 2. Example Western blots are shown on the *top* and densitometric quantification of three to four independent experiments *below*. Phosphorylated S6 was normalized to tubulin. Bar diagrams are means + SEM. None of the class IA PI3K catalytic subunits p110α, p110β, or p110δ was significantly different in S1 or S11 compared to their unaffected siblings (see Additional file 3: Figure S2)

We next assessed whether lymphoblastoid cell lines provide a suitable tool to identify molecular mechanisms leading to increased cell signaling. First, we performed Western blot analyses and confirmed that, similar as observed in the ELISA assays, S6 phosphorylation of cell line A4 (AGRE) was increased compared to cell line A21 and the unaffected control (Fig. 3a, b, one-way ANOVA *F*(2,9) = 35.36, *p* < 0.0001; Tukey’s post hoc analyses *p = 0.0004; ^#^*p* < 0.0001). We have previously shown that increased expression of the PI3K catalytic subunit p110β contributes to increased S6 phosphorylation in lymphoblastoid cell lines from patients with FXS [24]. We thus hypothesized that increased phospho-S6/S6 ratios in patient cell line A4 may be caused by increased expression and activity of a PI3K catalytic subunit. In contrast to FXS, the PI3K catalytic subunit p110β was not changed (Fig. 3a, c, one-way ANOVA *F*(2,9) = 0.66, *p* = 0.54). However, Western blot analyses revealed that cell line A4 had increased expression of the class I PI3K subunit p110δ compared to cell line A21 and the unaffected control (Fig. 3a, d, one-way ANOVA *F*(2,9) = 10.42, *p* = 0.0045; Tukey’s post hoc analyses *p = 0.0144; ^#^*p* = 0.0056). In line with the increased expression of p110δ, p110δ-associated PI3K activity was also elevated in cell line A4 compared to cell line A21 and the unaffected control (Fig. 3e, one-way ANOVA *F*(2,21) = 14.73, *p* = 0.0001; Tukey’s post hoc analyses *p = 0.0002, ^#^*p* = 0.0006). We further tested if similar defects were detectable in cell lines S1 and S11 (SSC; see Fig. 2). Western blot analyses showed increased S6 phosphorylation in S1 and S11 but not in S3 (Fig. 4, paired *t* tests, (a) *n* = 3, *t*(2) = 10.7, *p* = 0.009; (b) *n* = 4, *t*(3) = 10.39, *p* = 0.002; (c) *n* = 4, *t*(3) = 0.72, *p* = 0.525), thereby confirming our ELISA results (Fig. 2). In contrast to cell line A4, neither S1 nor S11 showed consistent and significant differences in the expression of the class IA PI3K subunits p110α, p110β, or p110δ (Additional file 3: Figure S2), suggesting that other upstream or downstream defects in the PI3K/mTOR pathway may cause altered S6 phosphorylation. Alternatively, mutations within PI3K catalytic subunits may change their activity but not expression and lead to increased S6 phosphorylation.

### Increased p110δ expression may be associated with autism diagnosis in a multiplex family

**Fig. 5:**
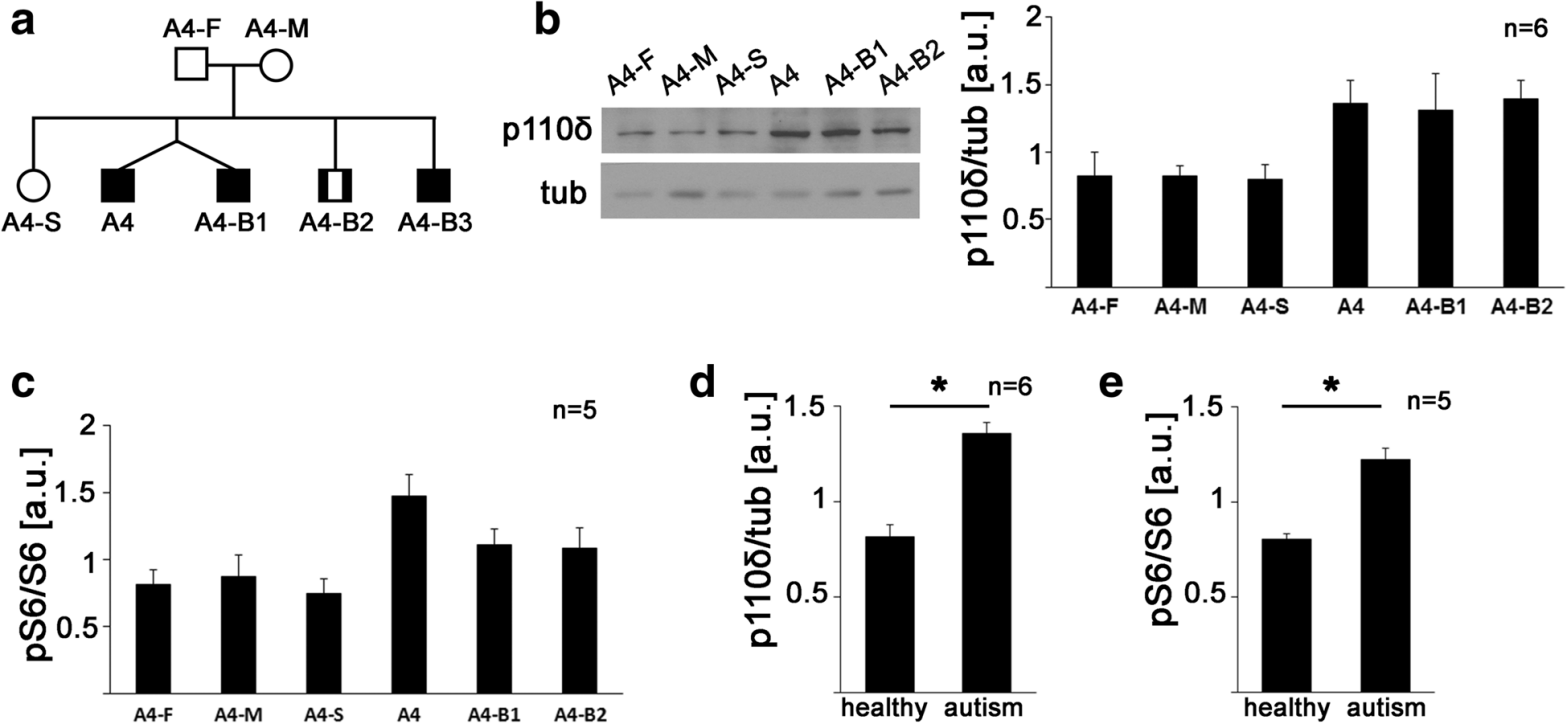
Increased p110δ expression and S6 phosphorylation is associated with autism in an autism multiplex family. **a** Pedigree of the multiplex family of patient A4. *Black* indicates autism diagnosis, and *black and white* indicates “not quite autism” (modified from the AGRE catalogue). **b** p110δ-specific Western blot analysis of the parents and three siblings of A4; example Western blot is shown on the *left* and quantification on the *right*. One-way ANOVA analysis shows a significant difference (*n* = 6, *F*(5,30) = 3.14, *p* = 0.021; *p* values of post hoc analyses comparing all family members to the healthy sibling shown in Additional file 4: Table S2a). **c** Similarly, phospho-S6-specific ELISAs suggested increased S6 phosphorylation in the autistic family members (*n* = 5, one-way ANOVA, *F*(5,24) = 3.77, *p* = 0.012; *p* values of post hoc analyses comparing all family members to the healthy sibling shown in Additional file 4: Table S2b). **d** Combining p110δ expression data from unaffected (A4-F, A4-M, A4-S) and autistic (A4, A4-B1, A4-B2) family members shows a significant increase in p110δ expression in the family members diagnosed with autism compared to their unaffected relatives (paired *t* test, *n* = 6, *t*(5) = 4.99, *p* = 0.004). **e** Combining phospho-S6 data from unaffected and autistic family members, respectively, again shows significantly increased S6 phosphorylation in cell lines from family members with autism (*n* = 5, paired *t* test, *t*(4) = 4.81, *p* = 0.009). Shown are means + SEM

We next took advantage of the fact that the AGRE collection stores lymphoblastoid cell lines and data from entire multiplex families. Cell line A4 was derived from an individual that has two unaffected parents (A4-F, A4-M), one unaffected sister (A4-S), and three brothers with autism (A4-B1, A4-B2, A4-B3), one of which is his twin brother (A4-B1) (Fig. 5a). Western blot analyses of the parents, the sister, and two brothers showed significant differences in p110δ expression among family members (Fig. 5b, one-way ANOVA, *F*(5,30) = 3.14, *p* = 0.021). Post hoc analyses comparing all family members to the healthy sister suggested that p110δ expression in the three affected sons was increased compared to the unaffected parents and sister, although statistical significance was not reached (*p* values for post hoc comparison to the unaffected sister shown in Additional file 4: Table S2a). Similarly, phospho-S6- and S6-specific ELISA analyses showed that phospho-S6/S6 ratios are significantly different among family members, and post hoc analyses suggested that phospho-S6/S6 ratios may be increased in the three family members with autism (Fig. 5c, one-way ANOVA, *F*(5,24) = 3.77, *p* = 0.012; *p* values for post hoc comparisons to the unaffected sister shown in Additional file 4: Table S2b). To further assess if increased p110δ expression and increased phosphorylation of S6 are associated with autism in this specific multiplex family, we compared pooled data from the autistic family members with those from unaffected family members. Analysis of pooled expression data from the three unaffected and the three affected lymphoblastoid cell lines showed that overall p110δ expression and S6 phosphorylation were significantly increased in lymphoblastoid cells from individuals with autism compared to unaffected individuals in this specific family (Fig. 5d, e, paired *t* tests, (d) *t*(5) = 4.99, *p* = 0.004; (e) *t*(4) = 4.81, *p* = 0.009). However, we cannot exclude that the difference observed in the pooled data, in particular in pS6/S6 ratios, was mainly driven by cell line A-4, which was originally identified in our screen. The cell line of one of the affected brothers (A4-B3) did not grow well and therefore was excluded from the analysis.

### Elevated S6 phosphorylation and protein synthesis rates in cell line A4 are reduced with a p110δ-selective inhibitor

**Fig. 6:**
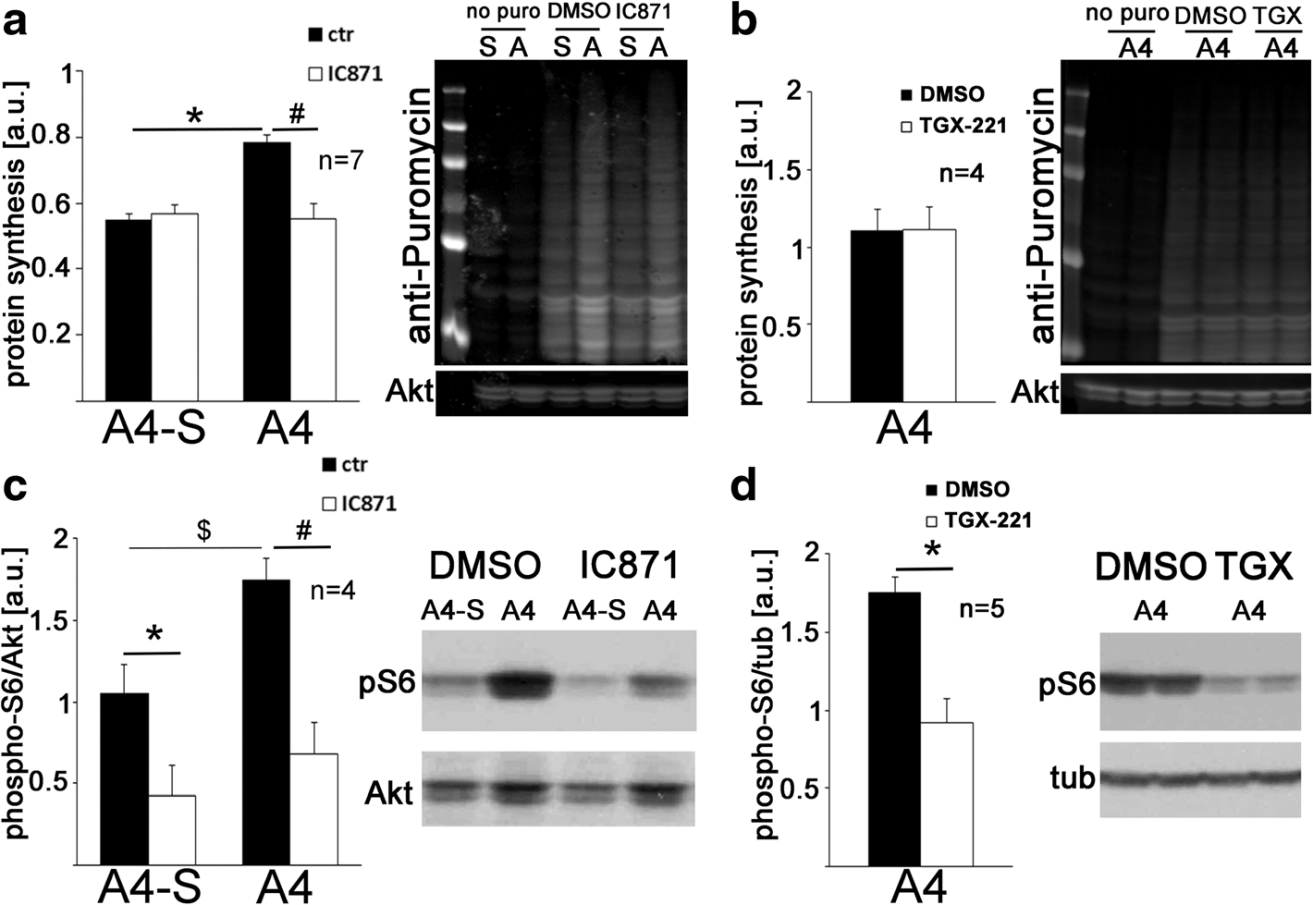
Increased S6 phosphorylation and protein synthesis in the patient cell line are decreased with a p110δ-selective inhibitor. **a** Protein synthesis assays using metabolic labeling of nascent peptide chains with puromycin followed by puromycin-specific Western blots show that basal protein synthesis rates in the cell line from the autistic patient (A4) are increased compared to cell lines from his unaffected sibling (A4-S). The p110δ-selective inhibitor IC87114 (1 nM, 15 min) reduces protein synthesis rates in the autistic patient but does not affect protein synthesis in the unaffected sibling (*n* = 7, two-way ANOVA *F*_interaction_(1,6) = 10.73, *p*_interaction_ = 0.017; *F*_treatment_(1,6) = 9.34, *p*_treatment_ = 0.022; *F*_autism_(1,6) = 1.20, *p*_autism_ = 0.316; Sidak’s post hoc analyses *p = 0.010, ^#^ *p* = 0.011). Example Western blot is shown on the *right*; “*no puro*” samples show background staining without puromycin treatment of the cells. **b** In contrast, the p110β-selective inhibitor TGX-221 (1 μM, 15 min) does not affect protein synthesis rates in cell line A4 (*n* = 4, paired *t* test, *t*(3) = 0.34, *p* = 0.76). Example Western blot is shown on the *right*; samples were loaded in duplicates. **c** Selective inhibition of p110δ with IC87114 decreases S6 phosphorylation in the cell lines from both the autistic individual and his unaffected sister. Quantification of ELISAs is shown on the *left* (*n* = 4, two-way ANOVA with Sidak’s post hoc tests *F*_autism_(1,3) = 35.01, *p*_interaction_ = 0.001; *F*_treatment_(1,3) = 163.2, *p*_treatment_ = 0.001; *F*_autism_(1,3) = 4.15, *p*_autism_ = 0.135; *p = 0.003, ^*#*^ *p* = 0.0005; ^$^ *p* = 0.0019). Example Western blot is shown on the *right*. **d** Similarly, selective inhibition of p110δ with TGX-221 reduces S6 phosphorylation in cell line A4 as measured by Western blotting (*n* = 5, paired *t* test, *t*(4) = 5.75, *p* = 0.005). Representative Western blot is shown on the *right*; samples were loaded in duplicates. Shown are means + SEM

Increased PI3K activity and S6 phosphorylation contribute to increased protein synthesis in FXS mouse models and cell lines from individuals with FXS [1, 24, 26–28, 33]. To assess whether elevated p110δ expression and activity in cell line A4 likewise leads to increased basal protein synthesis, we quantified protein synthesis rates in cell line A4 and the unaffected sister cell line A4-S using puromycin-labeling and anti-puromycin-specific Western blotting [25]. We detected increased protein synthesis rates in cell line A4, which were reduced to control levels by the p110δ-selective inhibitor IC87114 [34] (Fig. 6a, two-way ANOVA *F*_interaction_(1,6) = 10.73, *p*_interaction_ = 0.017; *F*_treatment_(1,6) = 9.34, *p*_treatment_ = 0.022; *F*_autism_(1,6) = 1.20, *p*_autism_ = 0.316; Sidak’s post hoc analyses *p = 0.010, ^#^*p* = 0.011). Protein synthesis rates in the cell line from the unaffected sibling were not affected by the p110δ inhibitor (*p* = 0.907). The specificity of the effect of the p110δ-inhibitor on protein synthesis rates was further confirmed by experiments showing that a selective inhibitor of the PI3K catalytic subunit p110β, TGX-221, did not significantly affect protein synthesis in cell line A4 (Fig. 6b, *n* = 4, paired *t* test, *t*(3) = 0.34, *p* = 0.76). In contrast, the p110δ-selective inhibitor reduced S6 phosphorylation in both cell lines, suggesting that additional p110δ-dependent mechanisms, apart from increased S6 phosphorylation, lead to increased protein synthesis rates in the cell line A4 (Fig. 6c, two-way ANOVA with Sidak’s post hoc tests *F*_autism_(1,3) = 35.01, *p*_interaction_ = 0.001; *F*_treatment_(1,3) = 163.2, *p*_treatment_ = 0.001; *F*_autism_(1,3) = 4.15, *p*_autism_ = 0.135; *p = 0.003, ^*#*^*p* = 0.0005; ^$^*p* = 0.0019). Of note, the p110β-selective inhibitor also reduced S6 phosphorylation in cell line A4 (Fig. 6d, *n* = 5, paired *t* test, *t*(4) = 5.75, *p* = 0.005), similarly as we have observed previously in lymphoblastoid cell lines from a patient with FXS and a healthy control [24].

## Discussion

The development of disease-modifying therapies for autism spectrum disorders is hindered by the variety of contributing factors and the limited knowledge about the underlying pathological mechanisms. Easily assessable biomarkers that offer insight into defective molecular pathways in individual patients provide a potential avenue for the design of targeted, personalized therapeutic strategies and could help to stratify patients into sub-cohorts that would benefit from certain treatments. Ideally, these biomarkers should provide quantitative measurements that can also be used to evaluate the efficiency of treatment strategies. Here, we provide initial evidence that quantitative screens for signaling defects in peripheral cell lines from individuals with autism could fulfill these criteria for a biomarker and are suitable to detect molecular pathological mechanisms that may serve as therapeutic targets. Apart from this conceptual advance, our results also corroborate previous studies suggesting that dysregulated PI3K/mTOR signaling is a common pathological mechanism shared by a sub-cohort of autism spectrum disorders with unknown etiology.

### Functional screens in peripheral blood cell lines are suitable to detect signaling defects in idiopathic autism

We used lymphoblastoid cell lines from patients with autism of unknown etiology obtained from the AGRE collection and the SSC to test the hypothesis that dysregulated PI3K/mTOR signaling is a shared pathological mechanism, treatment target, and biomarker in autism. This hypothesis is based on previous studies that found gene mutations in the PI3K/mTOR pathway in monogenic and idiopathic forms of autism [9, 12, 20]. Instead of screening for genetic defects, we took a functional approach that tested the activation of the PI3K/mTOR downstream target S6 in patient cells. This strategy has the advantage that molecular defects caused by a variety of factors, including genetic, epigenetic, and environmental influences, can be detected. In this pilot study, we screened lymphoblastoid cell lines from 58 individuals with autism and detected significantly increased S6 phosphorylation in 4 cell lines compared to a healthy control (AGRE cell lines) or their healthy siblings (SSC cell lines). This confirms previous genetic studies suggesting that defects in the PI3K/mTOR pathway may be a shared pathomechanism in a sub-cohort of individuals with autism [20], in addition to the known syndromic cases associated with altered PI3K/mTOR signaling (such as FXS, TS, and NF-1). Based on current published data, it is difficult to estimate what the prevalence of autism with PI3K/mTOR signaling defects is. Further complicating is the fact that it is unknown which of the observed autism-associated mutations within this pathway indeed lead to a functional defect and are thus truly disease-causing. To obtain a more accurate estimate of the percentage of autism spectrum disorders that have a PI3K/mTOR signaling defect detectable in peripheral cells, it will be essential to perform large-scale screens analyzing hundreds or thousands of individuals with autism. There are currently cell lines from ~2500 autistic patients and their siblings in the SSC, and ca. 1000 multiplex and simplex families in the AGRE collection, which could be analyzed in these functional screens in the future. Due to the relatively high variability of PI3K/mTOR signaling, we expect that only consistent and pronounced changes will be detectable, and some more moderate defects that might be disease-causing could be missed. In the future, it will be important to combine functional screens with genetic analyses to provide the highest sensitivity to detect alterations in the PI3K/mTOR pathway.

### Dysregulated signaling through p110δ in autism and schizophrenia suggests an important role in neuronal function

PI3K catalytic subunits exist in eight isoforms, which are categorized into class I, class II, and class III depending on their structure and function [35]. The four class I PI3K catalytic isoforms, p110α, p110β, p110γ, and p110δ, were shown to play distinct and unique roles in non-neuronal cells [36]. More recently, evidence is increasing that class I PI3K isoforms also have diverse functions in the brain and are dysregulated in neurological diseases [11]. For example, increased p110β expression was detected in the intellectual disability fragile X syndrome [1, 15, 24], and defects in p110δ expression and activity have been associated with schizophrenia [10]. In the present study, we show that p110δ is increased in several autistic siblings but not the unaffected sister of a multiplex family. This finding suggests that, apart from schizophrenia, altered p110δ is also implicated in the etiology of autism. We did not detect increased p110δ expression in two unrelated cell lines from autistic individuals with increased S6 phosphorylation, suggesting other upstream signaling defects. Future large-scale studies are needed to assess the prevalence of p110δ dysregulation in autism. However, even if the frequency is low, we believe that its usability as an easily detectable biomarker combined with the availability of PI3K subunit-selective drugs makes p110δ an attractive potential treatment target in autism.

So far, little is known about the molecular mechanisms regulated by p110δ in neurons. A previous study has shown that p110δ is important for axon outgrowth and regeneration [2], but the exact signaling pathways are unknown. Our study provides further insight into the signaling pathways regulated by p110δ by showing that increased p110δ expression in lymphoblastoid cells from an individual with autism leads to elevated S6 phosphorylation and increased protein synthesis rates, which can be reversed by a p110δ-selective inhibitor. Interestingly, while the p110δ-selective inhibitor reduced S6 phosphorylation in the cells from the autistic individual and his unaffected sister, it reduced protein synthesis rates only in cells from the autistic individual but not the unaffected sister. Notably, these results resemble our previous study, in which we made a very similar observation using a p110β-selective inhibitor in lymphoblastoid cell lines from an individual with fragile X syndrome [24]. Cells from individuals with FXS have increased p110β expression due to loss of translational control of the p110β messenger RNA (mRNA) [1, 24, 33]. A p110β-selective inhibitor reduced S6 phosphorylation in a cell line from an individual with FXS and in an unaffected control line, whereas protein synthesis rates were only reduced in cells from a patient with FXS but not altered by the inhibitor in control cells [24]. We speculate that both p110δ and p110β regulate S6 phosphorylation in lymphoblastoid cells, leading to reduced S6 phosphorylation when inhibited. In contrast, p110β- or p110δ-mediated protein synthesis may be a gain of function due to the increased p110β or p110δ levels in the FXS cells or in cell line A4, respectively. In line with this hypothesis, a selective p110β inhibitor reduced S6 phosphorylation in cell line A4 but did not affect protein synthesis, further supporting the specificity of the underlying defect in p110δ expression and activity in cell line A4.

Dysregulated mRNA translation was observed in several autism spectrum disorders [37], supporting the hypothesis that defects in p110δ-mediated protein synthesis may contribute to the autistic phenotype in this family and possibly others. We have recently shown that increased activity of p110β contributes to dysregulated protein synthesis downstream of group 1 metabotropic glutamate receptors in a mouse model of fragile X syndrome [28]. A previous study suggests that p110δ is signaling downstream of ErbB4 [10], but so far it is unknown if p110δ controls protein synthesis mediated by ErbB4 or other neurotransmitter receptors.

The fact that p110δ, which is dysregulated in schizophrenia, may be altered in autism further supports the hypothesis that autism spectrum disorders and schizophrenia share common neurobiology and contributing pathological mechanisms [38]. In non-neuronal cells, p110δ plays an important role in mediating inflammatory processes, and recently, p110δ has been proposed as a potential treatment target to reduce stroke-induced neuroinflammation in the brain [39]. Notably, both schizophrenia and autism spectrum disorders have been linked to increased neuroinflammation in the brain [40, 41]. In the future, it will be interesting to analyze the role of dysregulated p110δ activity in altered neuroinflammation in these diseases.

### Detecting functional molecular defects to identify therapeutic targets

A large number of studies have identified gene mutations or chromosomal CNVs that are associated with an increased risk to develop autism [42]. These studies have helped tremendously to shape our understanding of the diverse etiology of autism [43]. However, recent studies suggest a 50–60 % heritability of autism [44, 45], indicating that other factors in addition to gene mutations, such as the environment or epigenetics, play important roles in the development of autism. Most likely, autism and the contributing alterations on the molecular level arise from a combination of one or multiple gene mutations plus epigenetic factors and environmental influences. Here, we detected a functional defect; increased S6 phosphorylation caused by elevated expression of the PI3K subunit p110δ in autistic but not unaffected members of a multiplex autism family. Analyses of the available genetic data (AGRE) revealed no CNV affecting the *PIK3CD* gene (located on chromosome 1p36.2) in these families. Forthcoming studies will have to show if other genetic factors contribute to the observed signaling defects, for example, through targeted sequencing of candidate genes. In this study, we have found increased protein expression of p110δ. So far, the factors and signaling pathways influencing p110δ expression are mostly unknown [46, 47]. In the future, it will be interesting to reveal mechanisms regulating p110δ expression and to investigate if they are mutated in the multiplex family studied here. Analyzing the functional outcome of molecular defects in addition to genomic studies may help to advance the application of precision medicine to develop therapeutic treatments in individuals with autism. Functional studies also have the advantage to detect a molecular defect regardless of the contributing genetic, epigenetic, or environmental factors. This could facilitate the development of treatments targeted at a disease-causing defect without exact knowledge of the underlying cause.

## Conclusions

Using lymphoblastoid cell lines from patients with idiopathic autism, we confirm that defects in S6 phosphorylation occur in autism spectrum disorders of unknown etiology. For one case, we show that this defect is caused by alterations in PI3K/mTOR signaling, in particular the catalytic subunit p110δ. It is noteworthy that S6 phosphorylation is regulated by other signaling pathways, in addition to PI3K/mTOR, for example, the ERK1/2 pathway. The approach described here could thus detect alterations in several upstream signaling pathways, not only the PI3K/mTOR complex. Limitations of the analysis of lymphoblastoid cell lines are (1) that the process generating lymphoblastoid cell lines by Epstein-Barr virus-mediated transformation of blood lymphocytes may itself lead to alterations in signaling and (2) that defects that are restricted to neurons will not be detected. Artifacts in PI3K signaling that are due to the transformation of lymphocytes into lymphoblastoid cell lines could be avoided by directly analyzing lymphocytes purified from peripheral blood samples of patients. However, using fresh blood samples would make larger screens less feasible. Notably, our Western blot analyses suggest that p110δ expression is increased in lymphoblastoid cell lines from several autistic brothers within one family but not in lymphoblastoid cell lines from the unaffected sister or parents. These results suggest that the altered PI3K signaling is due to a genetic predisposition within this family and not an artifact caused by the process of lymphoblastoid cell generation. In the future, results generated with lymphoblastoid cells from patients should be confirmed in lymphocytes from peripheral blood samples. Analyzing lymphoblastoid cell lines is not suited to detect signaling defects that are restricted to neuronal cells; however, the feasibility of the method and the availability of large collections of lymphoblastoid cell lines from individuals with autism and their families justify this approach. In addition, previous studies in FXS have shown that brain defects detected in mouse models are also present in human peripheral cells, such as blood lymphocytes [32], lymphoblastoid cell lines [24], and fibroblasts [33]. In summary, the analysis of molecular signaling pathways in lymphoblastoid cell lines from patients with autism may help to identify potential treatment targets. More work is needed to assess if the molecular defect identified here, increased expression and activity of the PI3K catalytic subunit p110δ, could serve as a disease-modifying therapeutic target in autism.

### Ethics approval and consent to participate

The institutional review boards of Emory University and Cincinnati Children’s Hospital Medical Center determined that this research was “exempt” and no IRB approval was required. Therefore, no consent to participate was required. No animals were used for this research.

### Consent for publication

Not applicable.

## Additional files

Additional file 1: Figure S1a-c. Phospho-S6 and S6-specific ELISA results of independent repeats (*CTR(repeat)*) of the control shown in Fig. 1 (*CTR*) as well as one additional control cell line (*CTR-2*). No significant differences were detected in phospho-S6 levels (**a**, one-way ANOVA, *n* = 5–6, *F*(2,14) = 0.07, *p* = 0.94), S6-levels (**b**, one-way ANOVA, *n* = 5–6, *F*(2,14) = 0.49, *p* = 0.62), or pS6/S6 ratios (**c**, one-way ANOVA, *n* = 5–6, *F*(2,14) = 0.20, *p* = 0.82). **d** Comparison of all healthy siblings of the tested SSC cell lines (Fig. 2) shows that there was a significant difference (one-way ANOVA, *n* = 3–4, *F*(36,109) = 1.7, *p* = 0.018). This difference was driven by sibling S37 who had very low phospho-S6/S6 ratios, and when S37 was omitted, the *p* value was 0.18. Of note, this was an effect of the family S37, since S6 phosphorylation of the proband S37 was very low, too, and there was no difference between sibling S37 and the proband S37 (FDR-adjusted *p* = 0.919; Additional file 2: Table S1b). Shown are means + SEM. (TIF 407 kb) Additional file 2: Tables S1a and S1b. Complete list of *p* values for all paired comparisons made in Figs. 1 and 2. **a** List of *p* values of Dunnett’s post hoc analyses of each AGRE cell line compared to the healthy control. Shown are values for post hoc analyses for total S6, phosphorylated S6, and phospho-S6/S6 ratios. **b** List of false discovery rate-corrected *p* values of pairwise comparisons of phospho-S6/S6 ratios of SSC cell lines. Highlighted in yellow are comparisons with *p* < 0.05, which were considered significantly different. (DOC 66 kb) Additional file 3: Figure S2. Protein expression levels of PI3K catalytic subunits p110α (**a**), p110β (**b**), and p110δ (**c**) are unchanged in cell lines S1 and S11 compared to their healthy siblings. Representative Western blots are shown on the top, and densitometric quantifications of three to four separate experiments are on the bottom (paired *t* tests, **a**, S1: *n* = 4, *t*(3) = 1.75, *p* = 0.178; S11: *n* = 3, *t*(2) = 0.60, *p* = 0.607; **b**, S1: *n* = 4, *t*(3) = 1.79, *p* = 0.171; S11: *n* = 4, *t*(3) = 2.09, *p* = 0.128; **c**, S1: *n* = 4, *t*(3) = 0.79, *p* = 0.487; S11: *n* = 4, *t*(3) = 1.36, *p* = 0.266). Shown are means + SEM. (TIF 376 kb) Additional file 4: Tables S2a and S2b.
**a**, *p* values of Dunnett’s post hoc analyses of p110δ protein expression analyses of the multiplex family shown in Fig. 5b. **b**, *p* values of Dunnett’s post hoc analyses of phospho-S6/S6 ratios measured by ELISA of the multiplex family shown in Fig. 5c. All individual family members were compared to the unaffected sister A4-S. (DOC 7 kb)

## Abbreviations

AGRE: Autism Genetic Research Exchange
ANOVA: analysis of variance
CNV: chromosomal copy number variation
ELISA: enzyme-linked immunosorbent assay
FXS: fragile X syndrome
mTOR: mammalian target of rapamycin
NF1: neurofibromatosis 1
PI3K: phosphoinositide-3 kinase
PTEN: phosphatase and tensin homolog
SSC: Simons Simplex Collection
TS: tuberous sclerosis
