# Supplementary figures and images for: Increased expression of the PI3K catalytic subunit p110δ underlies elevated S6 phosphorylation and protein synthesis in an individual with autism from a multiplex family
Source: Mol Autism. 2016 Jan 14;7:3. doi: 10.1186/s13229-015-0066-4 (PMC4712554; doi:10.1186/s13229-015-0066-4)

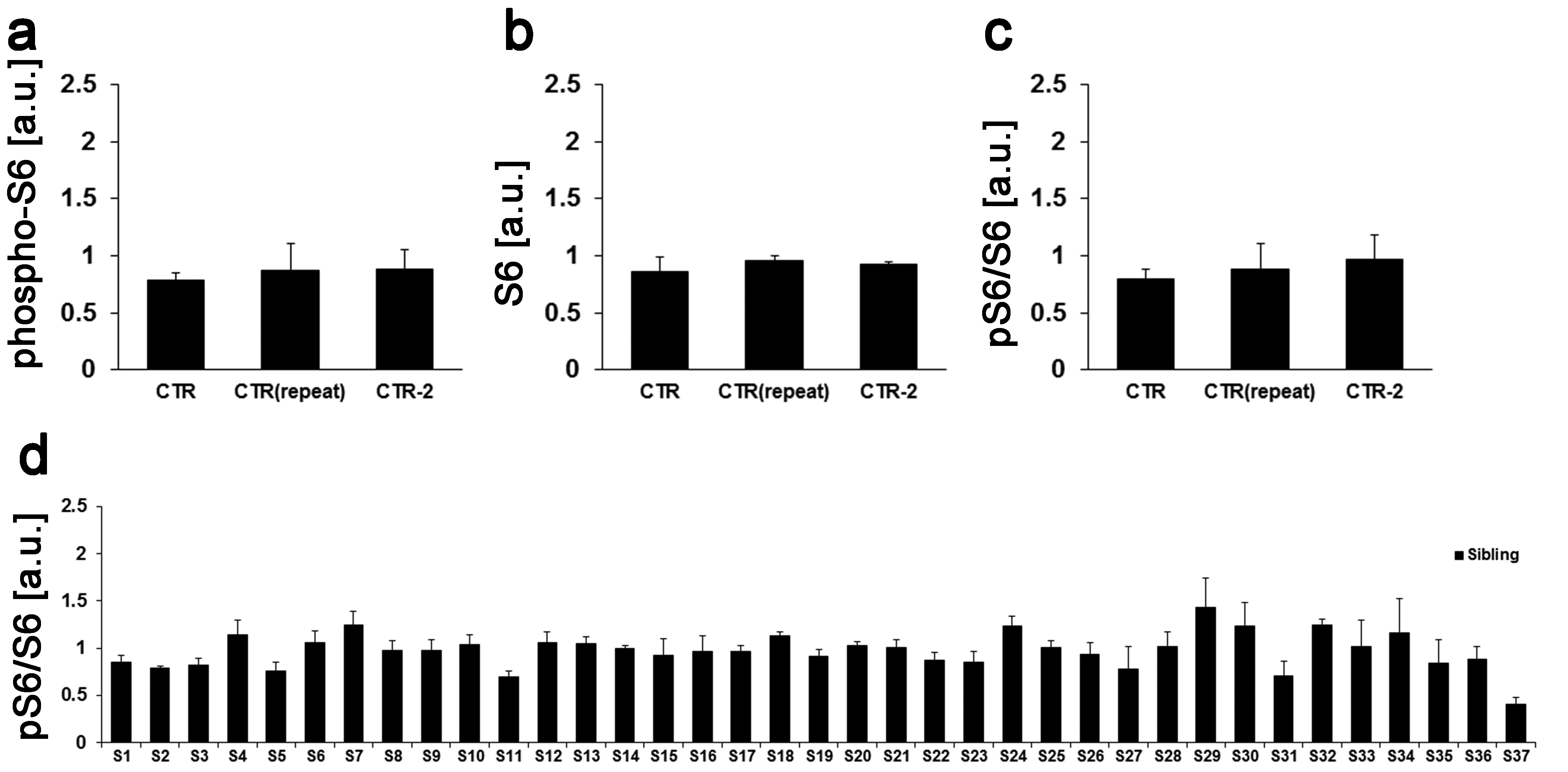

Supplement: Additional file 1: Figure S1a-c. — Phospho-S6 and S6-specific ELISA results of independent repeats (CTR(repeat)) of the control shown in Fig. 1 (CTR) as well as one additional control cell line (CTR-2). No significant differences were detected in phospho-S6 levels (a, one-way ANOVA, n = 5–6, F(2,14) = 0.07, p = 0.94), S6-levels (b, one-way ANOVA, n = 5–6, F(2,14) = 0.49, p = 0.62), or pS6/S6 ratios (c, one-way ANOVA, n = 5–6, F(2,14) = 0.20, p = 0.82). d Comparison of all healthy siblings of the tested SSC cell lines (Fig. 2) shows that there was a significant difference (one-way ANOVA, n = 3–4, F(36,109) = 1.7, p = 0.018). This difference was driven by sibling S37 who had very low phospho-S6/S6 ratios, and when S37 was omitted, the p value was 0.18. Of note, this was an effect of the family S37, since S6 phosphorylation of the proband S37 was very low, too, and there was no difference between sibling S37 and the proband S37 (FDR-adjusted p = 0.919; Additional file 2: Table S1b). Shown are means + SEM. (TIF 407 kb) [file 13229_2015_66_MOESM1_ESM.tif]

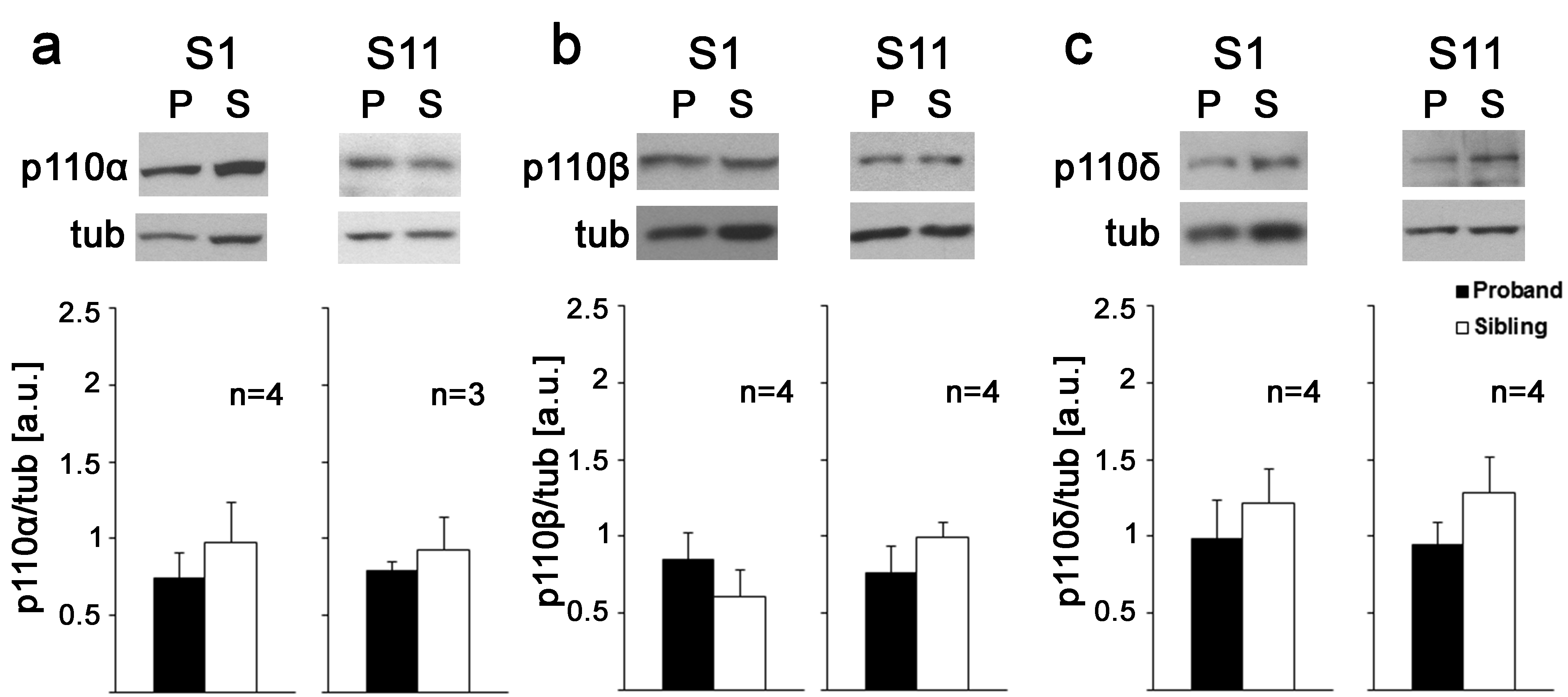

Supplement: Additional file 3: Figure S2. — Protein expression levels of PI3K catalytic subunits p110α (a), p110β (b), and p110δ (c) are unchanged in cell lines S1 and S11 compared to their healthy siblings. Representative Western blots are shown on the top, and densitometric quantifications of three to four separate experiments are on the bottom (paired t tests, a, S1: n = 4, t(3) = 1.75, p = 0.178; S11: n = 3, t(2) = 0.60, p = 0.607; b, S1: n = 4, t(3) = 1.79, p = 0.171; S11: n = 4, t(3) = 2.09, p = 0.128; c, S1: n = 4, t(3) = 0.79, p = 0.487; S11: n = 4, t(3) = 1.36, p = 0.266). Shown are means + SEM. (TIF 376 kb) [file 13229_2015_66_MOESM3_ESM.tif]
