# Supplementary material for: Increased expression of the PI3K catalytic subunit p110δ underlies elevated S6 phosphorylation and protein synthesis in an individual with autism from a multiplex family
Source: Mol Autism. 2016 Jan 14;7:3. doi: 10.1186/s13229-015-0066-4 (PMC4712554; doi:10.1186/s13229-015-0066-4)
Supplement: Additional file 2: Tables S1a and S1b. — Complete list of p values for all paired comparisons made in Figs. 1 and 2. a List of p values of Dunnett’s post hoc analyses of each AGRE cell line compared to the healthy control. Shown are values for post hoc analyses for total S6, phosphorylated S6, and phospho-S6/S6 ratios. b List of false discovery rate-corrected p values of pairwise comparisons of phospho-S6/S6 ratios of SSC cell lines. Highlighted in yellow are comparisons with p < 0.05, which were considered significantly different. (DOC 66 kb) [file 13229_2015_66_MOESM2_ESM.doc]

**Table S1a: p-values of Dunnett’s posthoc analyses of each AGRE cell line compared to the unaffected control**

| **cell line** | **p-S6** | **S6** | **pS6/S6** |
| --- | --- | --- | --- |
| **A1** | 0.0375 | 0.0127 | 0.3509 |
| **A2** | 0.9958 | 0.4941 | 0.9944 |
| **A3** | > 0.9999 | 0.9805 | > 0.9999 |
| **A4** | 0.0093 | 0.9457 | 0.0137 |
| **A5** | 0.0124 | 0.0003 | 0.3555 |
| **A6** | 0.9999 | 0.9951 | 0.9999 |
| **A7** | 0.9994 | 0.9993 | 0.9991 |
| **A8** | 0.9525 | 0.9992 | 0.9582 |
| **A9** | 0.9996 | 0.6508 | 0.9997 |
| **A10** | 0.9702 | 0.9877 | 0.8265 |
| **A11** | 0.9997 | 0.9994 | 0.9998 |
| **A12** | 0.9990 | 0.9996 | 0.9896 |
| **A13** | > 0.9999 | 0.2651 | 0.9998 |
| **A14** | 0.9991 | 0.2666 | > 0.9999 |
| **A15** | 0.9999 | 0.8368 | 0.9990 |
| **A16** | 0.9989 | 0.9994 | 0.9951 |
| **A17** | 0.7087 | 0.9996 | 0.7812 |
| **A18** | 0.9988 | 0.9992 | 0.9990 |
| **A19** | 0.9993 | 0.9789 | 0.9996 |
| **A20** | 0.6557 | > 0.9999 | 0.6989 |
| **A21** | 0.9997 | 0.9998 | 0.9997 |

**Table S1b: FDR-adjusted p-values of pairwise comparisons of proband and sibling cell lines from the Simons Simplex Collection**

| **cell line** | **pS6/S6** |
| --- | --- |
| **S1** | 0.003 |
| **S2** | 0.919 |
| **S3** | 0.919 |
| **S4** | 0.919 |
| **S5** | 0.919 |
| **S6** | 0.968 |
| **S7** | 0.919 |
| **S8** | 0.919 |
| **S9** | 0.968 |
| **S10** | 0.968 |
| **S11** | 0.508 |
| **S12** | 0.968 |
| **S13** | 0.968 |
| **S14** | 0.919 |
| **S15** | 0.919 |
| **S16** | 0.968 |
| **S17** | 0.919 |
| **S18** | 0.919 |
| **S19** | 0.919 |
| **S20** | 0.968 |
| **S21** | 0.919 |
| **S22** | 0.919 |
| **S23** | 0.919 |
| **S24** | 0.919 |
| **S25** | 0.919 |
| **S26** | 0.919 |
| **S27** | 0.919 |
| **S28** | 0.968 |
| **S29** | 0.919 |
| **S30** | 0.968 |
| **S31** | 0.919 |
| **S32** | 0.888 |
| **S33** | 0.919 |
| **S34** | 0.919 |
| **S35** | 0.919 |
| **S36** | 0.919 |
| **S37** | 0.919 |
