# Supplementary material for: Increased expression of the PI3K catalytic subunit p110δ underlies elevated S6 phosphorylation and protein synthesis in an individual with autism from a multiplex family
Source: Mol Autism. 2016 Jan 14;7:3. doi: 10.1186/s13229-015-0066-4 (PMC4712554; doi:10.1186/s13229-015-0066-4)
Supplement: Additional file 4: Tables S2a and S2b. — a, p values of Dunnett’s post hoc analyses of p110δ protein expression analyses of the multiplex family shown in Fig. 5b. b, p values of Dunnett’s post hoc analyses of phospho-S6/S6 ratios measured by ELISA of the multiplex family shown in Fig. 5c. All individual family members were compared to the unaffected sister A4-S. (DOC 7 kb) [file 13229_2015_66_MOESM4_ESM.doc]

**Table S2a: Dunnett’s posthoc analyses of p110δ protein**

| comparisons | p-values |
| --- | --- |
| A4-S <-> A4-F | 1 |
| A4-S <-> A4-M | 1 |
| A4-S <-> A4 | 0.106 |
| A4-S <-> A4-B1 | 0.138 |
| A4-S <-> A4-B2 | 0.074 |

**levels in the multiplex family described in Fig. 5.**

**Table S2b: Dunnett’s posthoc analyses of pS6/S6 ratios**

**in the multiplex family described in Fig. 5.**

| comparisons | p-values |
| --- | --- |
| A4-S <-> A4-F | 0.995 |
| A4-S <-> A4-M | 0.935 |
| A4-S <-> A4 | 0.004 |
| A4-S <-> A4-B1 | 0.213 |
| A4-S <-> A4-B2 | 0.298 |
